# Supplementary figures and images for: A prospective study of the adaptive changes in the gut microbiome during standard-of-care chemoradiotherapy for gynecologic cancers
Source: PLoS One. 2021 Mar 4;16(3):e0247905. doi: 10.1371/journal.pone.0247905 (PMC7932122; doi:10.1371/journal.pone.0247905)

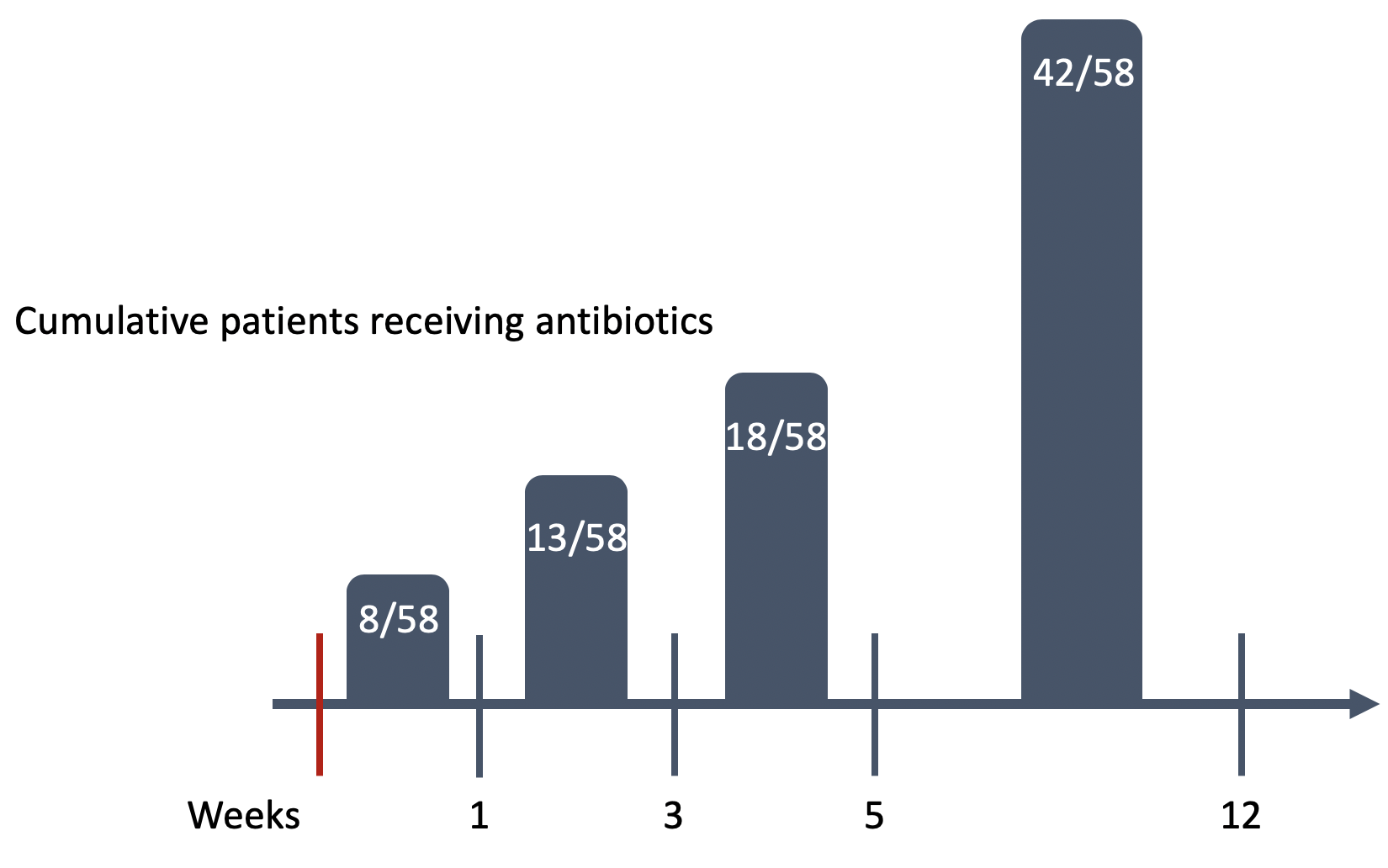

Supplement: S1 Fig — Number of patients who took antibiotics prior to each time point are presented. Total number of patients receiving antibiotics during the study period is 42. (TIF) [file pone.0247905.s001.tif]

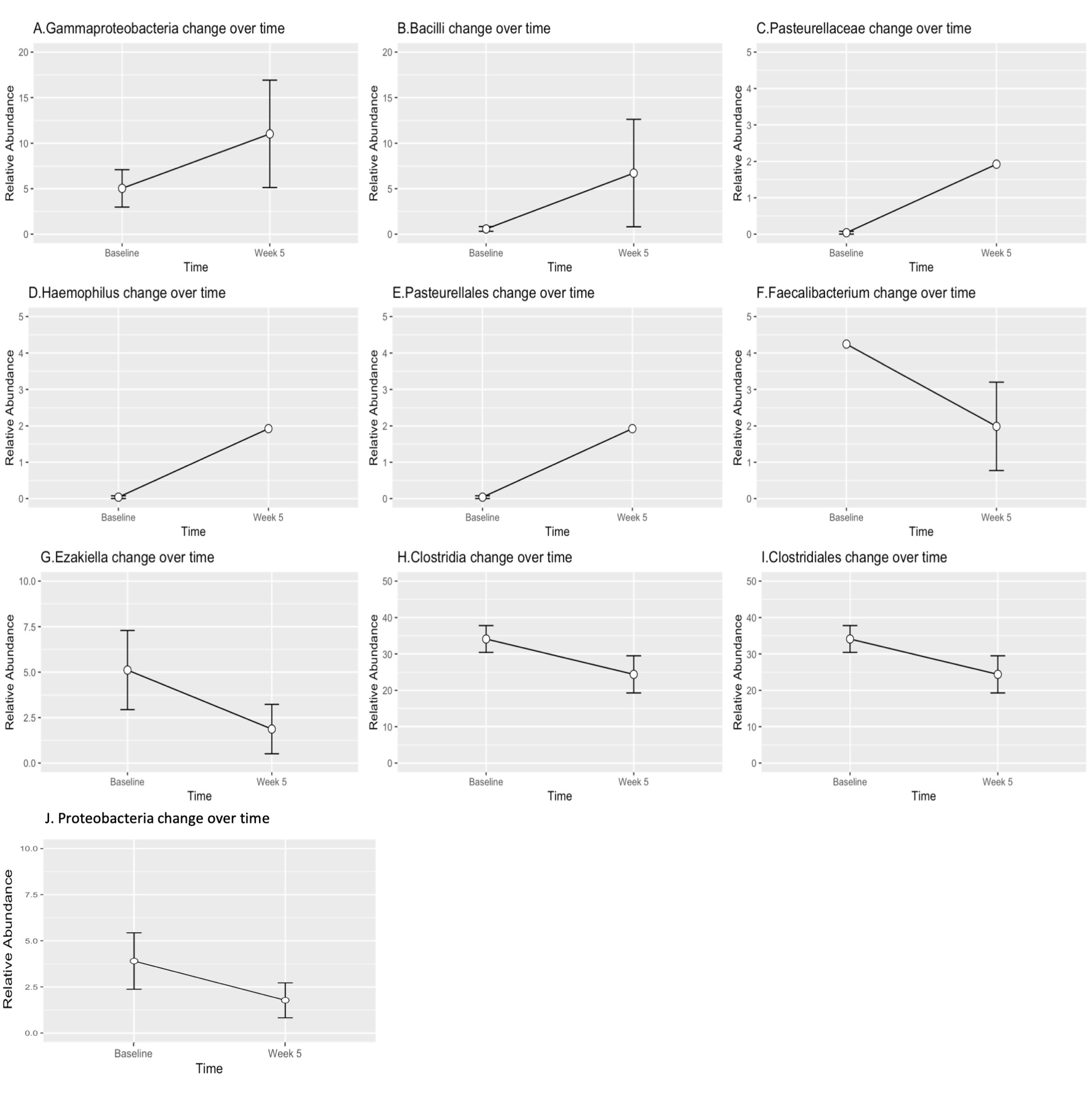

Supplement: S2 Fig — Relative abundances of species identified using linear discriminant analysis effect size as having changed between baseline and week 5. Whiskers on the plot represent the confidence interval. (TIF) [file pone.0247905.s002.tif]

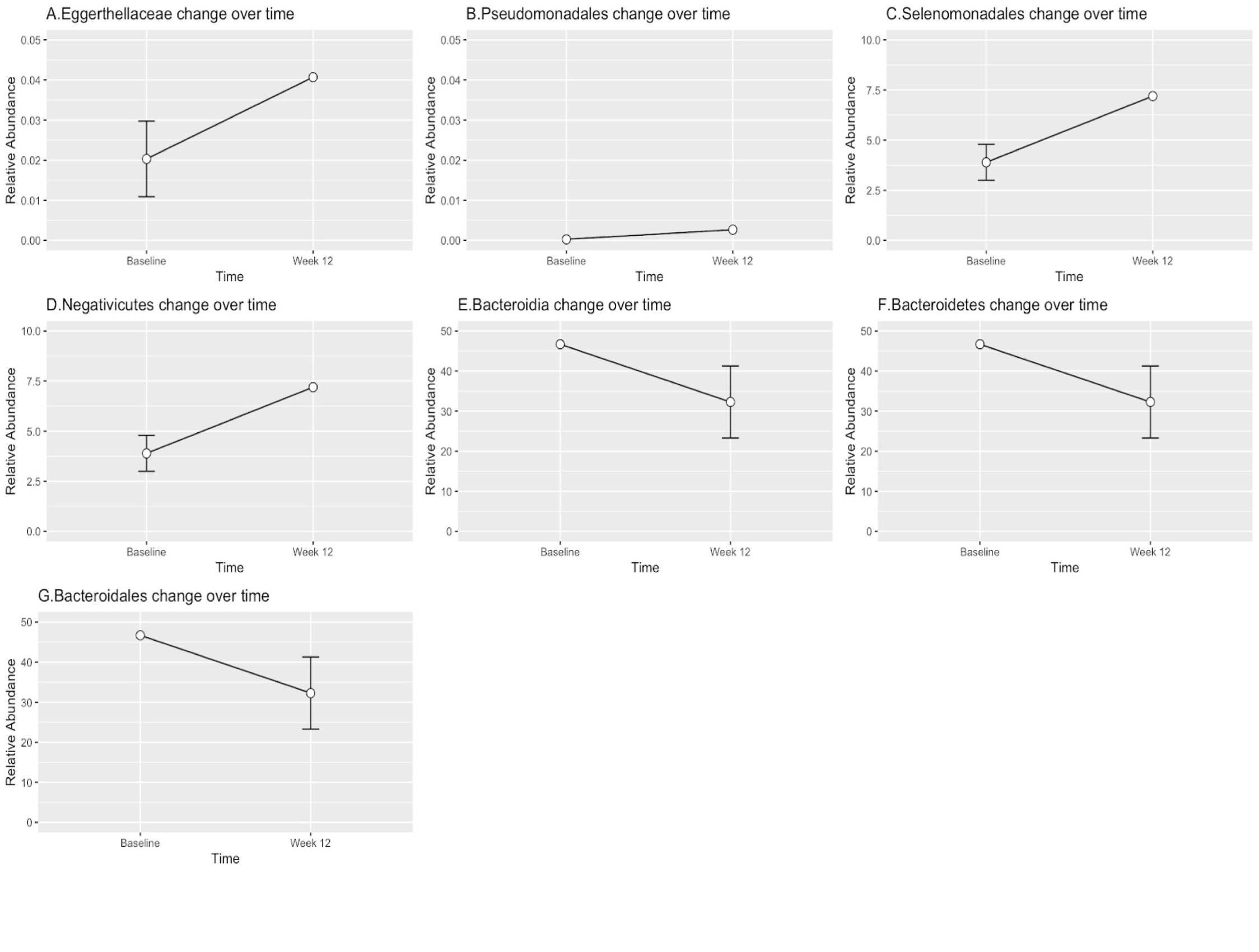

Supplement: S3 Fig — Relative abundances of species identified using linear discriminant analysis effect size as having changed between baseline and week 5 are plotted. Whiskers on the plot represent the confidence interval. (TIF) [file pone.0247905.s003.tif]
