# Supplementary material for: A prospective study of the adaptive changes in the gut microbiome during standard-of-care chemoradiotherapy for gynecologic cancers
Source: PLoS One. 2021 Mar 4;16(3):e0247905. doi: 10.1371/journal.pone.0247905 (PMC7932122; doi:10.1371/journal.pone.0247905)
Supplement: S1 Table — (DOCX) [file pone.0247905.s004.docx]

Supplemental Table 1. Range and average sequence sampling depth for each sample included in the study.

| **Patient #** | **Baseline**  Average (Range) | **Week 1**  Average (Range) | **Week 3**  Average (Range) | **Week 5**  Average (Range) | | **Week 12**  Average (Range) |
| --- | --- | --- | --- | --- | --- | --- |
| **1** | 7.77(0-2347) | 7.77(0-1388) | 7.77(0-2867) | – | | – |
| **2** | 7.77(0-3253) | – | – | – | | – |
| **3** | 7.77(0-1382) | 7.77(0-1633) | 7.77(0-1620) | 7.77(0-1227) | | – |
| **4** | 7.77(0-2601) | 7.77(0-1632) | 7.77(0-1478) | 7.77(0-3208) | | – |
| **5** | 7.77(0-3011) | – | – | – | | – |
| **6** | 7.77(0-3043) | 7.77(0-1898) | 7.77(0-2083) | 7.77(0-1521) | | – |
| **7** | 7.77(0-2256) | 7.77(0-1443) | 7.77(0-1592) | 7.77(0-4513) | | – |
| **8** | 7.77(0-1456) | 7.77(0-1370) | – | 7.77(0-2009) | | – |
| **9** | 7.77(0-4645) | – | – | 7.77(0-5225) | | – |
| **10** | 7.77(0-2845) | – | – | – | | – |
| **11** | 7.77(0-729) | 7.77(0-1735) | 7.77(0-1396) | 7.77(0-4787) | | – |
| **12** | 7.77(0-2288) | – | – | 7.77(0-3058) | | – |
| **13** | 7.77(0-1413) | 7.77(0-1163) | 7.77(0-1548) | 7.77(0-980) | | – |
| **14** | 7.77(0-1017) | 7.77(0-1964) | 7.77(0-2051) | 7.77(0-1839) | | – |
| **15** | 7.77(0-1328) | 7.77(0-1134) | 7.77(0-1024) | 7.77(0-3412) | | – |
| **16** | 7.77(0-1832) | 7.77(0-2325) | 7.77(0-1524) | 7.77(0-1648) | | – |
| **17** | 7.77(0-663) | 7.77(0-1149) | 7.77(0-1663) | 7.77(0-2921) | | – |
| **18** | 7.77(0-1862) | 7.77(0-3896) | – | – | | – |
| **19** | 7.77(0-1549) | 7.77(0-850) | 7.77(0-787) | 7.77(0-1314) | | – |
| **20** | 7.77(0-3195) | – | – | – | | – |
| **21** | 7.77(0-2226) | 7.77(0-1482) | 7.77(0-1445) | 7.77(0-1967) | | – |
| **22** | 7.77(0-2280) | 7.77(0-2496) | – | 7.77(0-4042) | | – |
| **23** | 7.77(0-1674) | 7.77(0-1278) | 7.77(0-2408) | 7.77(0-3167) | | – |
| **24** | 7.77(0-1971) | – | 7.77(0-1904) | 7.77(0-2009) | | – |
| **25** | 7.77(0-599) | – | 7.77(0-1141) | – | | – |
| **26** | 7.77(0-1311) | – | – | 7.77(0-1575) | | – |
| **27** | 7.77(0-1169) | 7.77(0-1284) | 7.77(0-2105) | 7.77(0-4043) | | – |
| **28** | 7.77(0-1114) | 7.77(0-2084) | 7.77(0-814) | 7.77(0-1945) | | – |
| **29** | 7.77(0-4310) | – | 7.77(0-1954) | 7.77(0-3096) | | – |
| **30** | 7.77(0-669) | 7.77(0-2081) | 7.77(0-996) | – | | 7.77(0-987) |
| **31** | 7.77(0-1487) | 7.77(0-2420) | 7.77(0-1323) | 7.77(0-1761) | | – |
| **32** | 7.77(0-1733) | 7.77(0-1633) | – | 7.77(0-2344) | | 7.77(0-1887) |
| **33** | 7.77(0-853) | – | 7.77(0-1202) | 7.77(0-709) | | 7.77(0-980) |
| **34** | 7.77(0-2475) | 7.77(0-1915) | 7.77(0-3895) | 7.77(0-3797) | | 7.77(0-1029) |
| **35** | 7.77(0-1483) | 7.77(0-983) | 7.77(0-1442) | | – | 7.77(0-1151) |
| **36** | 7.77(0-1850) | 7.77(0-1929) | 7.77(0-958) | 7.77(0-785) | | 7.77(0-1328) |
| **37** | 7.77(0-1209) | – | – | 7.77(0-1781) | | – |
| **38** | 7.77(0-1663) | 7.77(0-2099) | – | 7.77(0-2114) | | – |
| **39** | 7.77(0-2060) | – | – | 7.77(0-6318) | | 7.77(0-1972) |
| **40** | 7.77(0-1462) | 7.77(0-931) | – | 7.77(0-3322) | | – |
| **41** | 7.77(0-1541) | 7.77(0-1526) | 7.77(0-1206) | 7.77(0-1934) | | 7.77(0-3373) |
| **42** | 7.77(0-1192) | 7.77(0-1572) | 7.77(0-1514) | 7.77(0-1139) | | 7.77(0-2266) |
| **43** | – | – | – | 7.77(0-3088) | | – |
| **44** | 7.77(0-1301) | 7.77(0-1084) | 7.77(0-2099) | – | | 7.77(0-1103) |
| **45** | 7.77(0-1457) | 7.77(0-1506) | 7.77(0-1489) | – | | 7.77(0-1738) |
| **46** | 7.77(0-1371) | 7.77(0-1521) | 7.77(0-1079) | 7.77(0-1651) | | 7.77(0-3807) |
| **47** | 7.77(0-900) | – | 7.77(0-599) | 7.77(0-1393) | | 7.77(0-1428) |
| **48** | 7.77(0-1166) | – | – | 7.77(0-3311) | | 7.77(0-2374) |
| **49** | 7.77(0-1331) | – | – | – | | – |
| **50** | – | 7.77(0-1584) | 7.77(0-3510) | 7.77(0-1386) | | 7.77(0-1189) |
| **51** | 7.77(0-918) | – | – | 7.77(0-1173) | | 7.77(0-1013) |
| **52** | 7.77(0-2485) | 7.77(0-1901) | 7.77(0-982) | – | | – |
| **53** | 7.77(0-1809) | 7.77(0-1626) | 7.77(0-830) | 7.77(0-1112) | | – |
| **54** | 7.77(0-944) | 7.77(0-946) | – | – | | – |
| **55** | 7.77(0-2092) | 7.77(0-3725) | – | 7.77(0-1920) | | – |
| **56** | – | – | 7.77(0-991) | – | | – |
| **57** | 7.77(0-3742) | – | – | – | | – |
| **58** | 7.77(0-643) | 7.77(0-1296) | 7.77(0-936) | 7.77(0-1208) | | – |
