# Supplementary material for: A prospective study of the adaptive changes in the gut microbiome during standard-of-care chemoradiotherapy for gynecologic cancers
Source: PLoS One. 2021 Mar 4;16(3):e0247905. doi: 10.1371/journal.pone.0247905 (PMC7932122; doi:10.1371/journal.pone.0247905)
Supplement: S2 Table — (DOCX) [file pone.0247905.s005.docx]

**Supplemental Table 2.** **Patient demographic and clinical characteristics (N=58)**

|  | N | (%) |
| --- | --- | --- |
| **Mean age** (SD) | 49.36 (10.52) | — |
| **BMI**, Mean (SD), kg/m2 | 28.7(6.07) | — |
| **Type of Cancer** | | |
| Cervical | 55 | (94.8) |
| Vaginal | 2 | (3.4) |
| Vulvar | 1 | (1.7) |
| **Race/Ethnicity** | | |
| Asian | 2 | (3.5) |
| Black | 4 | (7.0) |
| Hispanic | 26 | (45.6) |
| White | 24 | (42.1) |
| Other | 1 | (1.8) |
| **FIGO Stage** | | |
| IA1 | 1 | (1.7) |
| IA2 | 0 | (0) |
| IB1 | 6 | (10.3) |
| IB2 | 6 | (10.3) |
| IIA | 3 | (5.2) |
| IIB | 28 | (48.3) |
| IIIA | 9 | (15.5) |
| IIIB | 0 | (0) |
| IVA | 4 | (6.9) |
| IVB | 1 | (1.7) |
| **Grade** | | |
| Well | 3 | (5.2) |
| Moderate | 20 | (34.5) |
| Poor | 26 | (44.8) |
| Unknown | 9 | (15.5) |
| **Histology** | | |
| Squamous | 46 | (79.3) |
| Adenocarcinoma | 10 | (17.2) |
| Adenosquamous | 2 | (3.4) |
| **Node Level on PET** | | |
| Common Iliac | 9 | (15.5) |
| External Iliac | 23 | (39.7) |
| Internal Iliac | 6 | (10.3) |
| Para-Aortic | 3 | (5.2) |
| None | 17 | (29.3) |
| **Smoking status** | | |
| Current | 5 | (8.8) |
| Former | 21 | (36.8) |
| Never | 31 | (54.4) |
| **Antibiotic Use** | | |
| No | 16 | (27.6) |
| Yes | 42 | (72.4) |
| **Brachytherapy** | | |
| HDR | 21 | (38.2) |
| PDR | 34 | (61.8) |
| **HPV Status** | | |
| Positive | 45 | (77.6) |
| Negative | 10 | (17.2) |
| Unknown | 3 | (5.2) |
