# Supplementary material for: A prospective study of the adaptive changes in the gut microbiome during standard-of-care chemoradiotherapy for gynecologic cancers
Source: PLoS One. 2021 Mar 4;16(3):e0247905. doi: 10.1371/journal.pone.0247905 (PMC7932122; doi:10.1371/journal.pone.0247905)
Supplement: S3 Table — (DOCX) [file pone.0247905.s006.docx]

**Supplemental Table 3. Samples available for each patient at each time point.**

| **Patient #** | **Baseline** | **Week 1** | **Week 3** | **Week 5** | **Week 12** |
| --- | --- | --- | --- | --- | --- |
| **1** |  |  |  |  |  |
| **2** |  |  |  |  |  |
| **3** |  |  |  |  |  |
| **4** |  |  |  |  |  |
| **5** |  |  |  |  |  |
| **6** |  |  |  |  |  |
| **7** |  |  |  |  |  |
| **8** |  |  |  |  |  |
| **9** |  |  |  |  |  |
| **10** |  |  |  |  |  |
| **11** |  |  |  |  |  |
| **12** |  |  |  |  |  |
| **13** |  |  |  |  |  |
| **14** |  |  |  |  |  |
| **15** |  |  |  |  |  |
| **16** |  |  |  |  |  |
| **17** |  |  |  |  |  |
| **18** |  |  |  |  |  |
| **19** |  |  |  |  |  |
| **20** |  |  |  |  |  |
| **21** |  |  |  |  |  |
| **22** |  |  |  |  |  |
| **23** |  |  |  |  |  |
| **24** |  |  |  |  |  |
| **25** |  |  |  |  |  |
| **26** |  |  |  |  |  |
| **27** |  |  |  |  |  |
| **28** |  |  |  |  |  |
| **29** |  |  |  |  |  |
| **30** |  |  |  |  |  |
| **31** |  |  |  |  |  |
| **32** |  |  |  |  |  |
| **33** |  |  |  |  |  |
| **34** |  |  |  |  |  |
| **35** |  |  |  |  |  |
| **36** |  |  |  |  |  |
| **37** |  |  |  |  |  |
| **38** |  |  |  |  |  |
| **39** |  |  |  |  |  |
| **40** |  |  |  |  |  |
| **41** |  |  |  |  |  |
| **42** |  |  |  |  |  |
| **43** |  |  |  |  |  |
| **44** |  |  |  |  |  |
| **45** |  |  |  |  |  |
| **46** |  |  |  |  |  |
| **47** |  |  |  |  |  |
| **48** |  |  |  |  |  |
| **49** |  |  |  |  |  |
| **50** |  |  |  |  |  |
| **51** |  |  |  |  |  |
| **52** |  |  |  |  |  |
| **53** |  |  |  |  |  |
| **54** |  |  |  |  |  |
| **55** |  |  |  |  |  |
| **56** |  |  |  |  |  |
| **57** |  |  |  |  |  |
| **58** |  |  |  |  |  |
