# Supplementary material for: A prospective study of the adaptive changes in the gut microbiome during standard-of-care chemoradiotherapy for gynecologic cancers
Source: PLoS One. 2021 Mar 4;16(3):e0247905. doi: 10.1371/journal.pone.0247905 (PMC7932122; doi:10.1371/journal.pone.0247905)
Supplement: Attachment — Submitted filename: Response to Reviewers.docx [file pone.0247905.s008.docx]

Manuscript ID: PONE-D-20-16939R1

Title: Adaptive changes in the gut microbiome during standard-of-care chemoradiotherapy for gynecologic cancers

Dear PLOS ONE Reviewers,

We are very excited to have been given a second opportunity to revise our manuscript entitled “Adaptive changes in the gut microbiome during standard-of-care chemoradiotherapy for gynecologic cancers” for PLOS ONE. We want to extend our appreciation for taking the time and effort necessary to provide such insightful guidance. Herein, we explain how we revised the paper based on your comments and recommendations.

*Note:* Page and line numbers refer to the revised manuscript with track changes

**EDITOR, COMMENT 1**

*Raw data should be deposited in a public repository (e.g., SRA).*

**Response:**

We have deposited our raw data in the Sequence Read Archive (SRA). The project accession number is **PRJNA685389**

**REVIEWER 3, COMMENT 1 (Title)**

*Consider adding “prospective” to the title so as to better frame your study design for readers. For example, the title might read, “Prospective study of adaptive changes in the gut microbiome…”*

**Response:**

We appreciate the reviewer’s suggestion. We have changed the title to “A prospective study of the adaptive changes in the gut microbiome during standard-of-care chemoradiotherapy for gynecologic cancers”

**REVIEWER 3, COMMENT 2 (Methods)**

*All eligible patients who were seen in clinic were given the opportunity to participate in the study. 58 individuals consented. What was the total number of patients who were approached? This should be stated in the Materials and Methods. Were there any significant differences between those who agreed to participate in the study and those who did not?*

**Response:**

We appreciate the reviewer’s comment. In our study, every eligible patient was approached to be included in our study, however some patients refused to participate since they did not want additional tissue samples taken. We are not sure of the exact number of patients approached during this study period, but our clinic sees on average 75 patients per year. We have added “Our clinic sees an average of 75 patients per year.” in the methods section.

-Edits to the manuscript can be found on page 6, lines 83-84

**REVIEWER 3, COMMENT 3 (Abstract)**

*It is important to include in the abstract the number of subjects who received antibiotics during the study period. This represents a major confounder and should be rightfully mentioned in the summary.*

**Response:**

We thank the reviewer for their suggestion. We have included the number of subjects receiving antibiotics during the study in the abstract. “42 of these patients received antibiotics during the study period.”

- Edits can be found on page 3, lines 17-18.

**REVIEWER 3, COMMENT 4 (Methods)**

*Line 125, “For each patient who provided samples at all five time points (n=17)…” – only 16 samples were counted in Table S2. Please reconcile.*

**Response:**

We appreciate the reviewer’s comment. For this specific analysis used for figure 2, we used data from patients who had samples at all three timepoints (baseline, week 5, and week 12). This seems to have been a typo, thank you for your attention to detail. We have revised this sentence and now reads “for each patient who provided samples at baseline, week 5, and week 12 (n=11), we tracked the changes in diversity over time to determine the number of patients for whom gut diversity returned to baseline levels.”

- Edits to the manuscript can be found on page 8, lines 127-130.

**REVIEWER 3, COMMENT 5 (Methods)**

*Lines 127-128, “for patients who did not provide samples at all time points (n=58)” – should not this be 16 (or 17)? Please clarify.*

**Response:**

We thank the reviewer for their comment. We built a linear mixed model accounting for patients who had missing data for one or more timepoints. In our study, we had 53 patients with at least one missing time point and so we have corrected the sentence and now reads “For patients who did not provide samples at all time points (n=53), we built a linear mixed model for time as a predictor of diversity”

- Edits to the manuscript can be found on page 8, line 133

**REVIEWER 3, COMMENT 6 (Methods)**

*Please provide the range and average sequence sampling depth for all samples included in the study. Sufficient sampling depth is needed so that minor OTUs are not overlooked. The authors should assure that OTUs were assessed with >95% confidence at, for example, a relative abundance of >0.1% or >0.01%. Sufficient sequence sampling depth is needed to minimize artificial skewing of diversity assessments.*

**Response:**

We thank the reviewer for their suggestion. We have added the range and mean sequence sampling depth for all samples included in the study in a new Supplemental Table 1. We have also included the following statement in the methods section: “The range and average sequence sampling depth for each sample is provided in S1 Table.” We have also mentioned in the text that the OTU assignment was performed with 97% sequence similarity.

- Edits to the manuscript can be found on page 8, lines 117-118; and pages 30-31, lines 531-533

**REVIEWER 3, COMMENT 7 (Discussion)**

*So few samples were collected at week 12 (16 [17%] out of 58 initially enrolled subjects) that any comparison to baseline samples is difficult to interpret (lines 199 – 215). It cannot be known how biases may have been introduced by dropouts that subsequently affected comparisons. This limitation was not adequately discussed. Frankly, the stark attrition at week 12 suggested to this reviewer that results for week 12 as shown in Table 1 and Fig. 2, if included at all, should be relegated to supplemental data, as was the case with supplemental Fig. 3, and caveats included in the text about the limitations to any interpretation.*

**Response:**

We appreciate the reviewer’s comment. We have tried to be very careful with our conclusions regarding data at week 12. Although a high attrition rate in studying is of valuable concern, our study is a longitudinal one and so patients were compared to themselves using paired statistical tests. We have removed the results of week 12 in Table one and have included the following sentence to the results section. “Diversity at week 12 was also compared to baseline, however we have not included these results in Table 1 due to a low patient number at this time point.”. We would prefer to keep Figure 2 as a visualization but have taken care to not present any statistical tests. The aim of the figure is to visualize and trace the diversity over time in patients who had provided samples for all three time points. We have clarified this point in the figure legend and text.

-Edits to the manuscript can be found on page 11, lines 194-195
